# *EGFR*基因突变与肺腺癌主要病理分型及标本类型的关系

**DOI:** 10.3779/j.issn.1009-3419.2017.06.03

**Published:** 2017-06-20

**Authors:** 丽菲 康, 杰 郑, 翔 朱

**Affiliations:** 1 100191 北京，北京大学医学部病理学系 Department of Pathology, Peking University, Beijing 100191, China; 2 050041 石家庄，河北省胸科医院病理科 Department of Pathology, Hebei Chest Hospital, Shijiazhuang 050041, China

**Keywords:** 肺腺癌, EGFR, 手术切除标本, 活检标本, Lung adenocarcinoma, EGFR, Surgical removal specimen, Biopsy specimen

## Abstract

**背景与目的:**

随着基因突变技术及靶向药物治疗如火如荼的开展，对肺腺癌的精准治疗越来越受到关注，目前肺腺癌中研究最多的是表皮生长因子受体（epidermal growth factor receptor, EGFR）。对于*EGFR*突变和病理分型的关系在不同标本中是否一致，目前不甚明了。本研究比较肺腺癌活检标本和手术切除标本中*EGFR*基因突变与病理分型的关系是否一致，探讨*EGFR*基因突变与肺腺癌病理分型的关系以及标本类型对*EGFR*基因检测的影响。

**方法:**

收集肺腺癌手术切除标本（楔形肺切除、肺叶切除标本）163例，肺腺癌活检[粘膜活检、肺穿刺、支气管内超声引导针吸活检术（endobronchial ultrasound-guided transbronchial needle aspiration, EBUS-TBNA）标本]173例，按照2015年世界卫生组织（World Health Organization, WHO）肺腺癌分型标准对其主要组织学分型确认（贴壁型、腺泡型、乳头型、微乳头型、实体型），行*EGFR*基因检测[基因测序法及突变扩增阻滞系统（amplification refractory mutation system, ARMS）]。分别对活检标本和手术切除标本进行统计。

**结果:**

163例的肺腺癌手术切除标本中，102例*EGFR*基因突变，突变率为62.58%，173例的活检标本中，114例*EGFR*基因突变，突变率为65.9%。两组标本中*EGFR*突变率没有统计学差异（*P* > 0.05）。两组标本中女性的*EGFR*突变率均明显高于男性（*P* < 0.05）。手术切除标本中60岁以上患者的*EGFR*突变率明显低于60岁以下（*P* < 0.05），而活检标本中*EGFR*突变与年龄无关（*P* > 0.05）。在*EGFR*突变的两组标本中病理分型构成比不同（χ^2^=8.040, *P* < 0.05）。手术切除标本肺腺癌中*EGFR*突变的102例中，腺泡型占54.9%（56例），贴壁型占23.53%（24例），乳头型占17.65%（18例），实体型占3.9%（4例），其中腺泡型所占比例最高，其次是贴壁型和乳头型，实体型则比例最少。19、21外显子单独突变最多，21外显子突变在贴壁型较其他两型高（*P* < 0.05），19外显子突变在乳头型较贴壁型高（*P* < 0.05）。腺泡型和乳头型比较，19、21外显子突变无统计学意义。活检标本肺腺癌中*EGFR*突变的114例中腺泡型占48.25%（55例），贴壁型占26.32%（30例），乳头型占11.4%（13例），微乳头型占4.39%（5例），实体型占9.65%（11例）。腺泡型所占比例最高，其次是贴壁型，乳头状、微乳头状和实体型最少。同样是19、21外显子单独突变最多，但不同病理分型中，19、21外显子突变均无显著差异（*P* > 0.05）。

**结论:**

肺腺癌中手术切除标本和活检标本*EGFR*突变率没有差异，且突变与性别有关，均为女性突变率高于男性。手术切除标本中*EGFR*突变与年龄有关，年龄越大突变率越低，而在活检标本中则与年龄无关。两组标本的病理分型构成比不同。19、21外显子单独突变最多。手术切除标本中*EGFR*突变类型与主要病理分型有关，21外显子单独突变中贴壁型最多，19外显子单独突变中乳头型最多。*EGFR*突变活检标本中，19、21外显子单独突变与主要病理分型无明显相关。

## Abstract (en)

**Background and objective:**

With the development of genetic mutations and targeted drugs, accurate therapy of lung adenocarcinoma attracts much more attention, and more research is focued on epidermal growth factor receptor (EGFR). It is unclear whether the result of *EGFR* mutation and pathology type is consistent with different specimens. In our study, by comparing the relationship between *EGFR* mutations and pathological classification of lung adenocarcinoma in surgical resection of specimen and biopsy specimen, to discuss the relationship between *EGFR* mutations and pathological classification of and the influence of specimen type on *EGFR* gene detection.

**Methods:**

A total of 163 cases of surgical resection of sample of lung adenocarcinoma (pulmonary resection and pulmonary lobectomy) and 173 cases of biopsy specimen [mucosa biopsy, needle biopsy of lung, and endobronchial ultrasound-guided transbronchial needle aspiration (EBUS-TBNA)] were performed by gene sequencing method and amplification refractory mutation system (ARMS) and the majority of the type was confirmed (lepidic, acinar, papillary, micropapillary, solid) according to the classification of lung adenocarcinoma in 2015 World Health Organization (WHO). The statistics was used in surgical and biopsy sample respectively.

**Results:**

The gene mutation of *EGFR* in surgical and biopsy sample of lung adenocarcinoma was 62.58% (102/163) and 65.9% (114/173) respectively, and no significant difference was found (*P* > 0.05). The mutation of *EGFR* in female was predominant both of the two groups (*P* < 0.05). The mutation rate of *EGFR* over the age of 60 was significantly lower than that below 60 in surgical specimen, while it was not related to age in biopsy sample. The constituent ratio of pathology type was different in the two groups (χ^2^=8.04, *P* < 0.05). Among 102 cases of lung adenocarcinoma in surgical specimen, the acinar took up the highest proportion (54.9%), followed by the lepidic (23.53%) and the papillary (17.65%). The solid adenocarcinoma accounted for the minimal percentage (3.9%). The mutation of 19 and 21 exon alone was most common. The mutation rate of 21 exon in the lepidic was higher than that in the acinar and papillary (*P* < 0.05), but the mutation rate of 19 exon in the papillary was higher than that in the lepidic (*P* < 0.05). There was no significant difference of 19 and 21 exon in the acinar and papillary. Among 114 cases of lung adenocarcinoma in the biopsy specimen, the most percentage was the acinar (48.25%), the lepidic was secondly, and the papillary, micropapillary and solid adenocarcinoma was the minimal. The exon mutation of 19 and 21 exon alone was most common, while no obvious difference of 19 and 21 exon was found in different pathology classifications (*P* > 0.05).

**Conclusion:**

The mutation rate of *EGFR* of lung adenocarcinoma in surgical resected specimen and biopsy specimen was not found difference, which was related to sex, and the female was predominant. The mutation rate of surgical specimen was higher in the young, while that of biopsy specimen was not related to the age. Apparent difference of the pathology type proportion was found in the two groups. The mutation of 19 and 21 exon alone was most common. The mutation of *EGFR* in surgical specimens was related to pathology types. The percentage of the lepidic adenocarcinoma was highest in the mutation of 21 exon alone. Among the mutation of 19 exon alone, the papillary was predominant. There was no obvious relationship between the mutation of 19 and 21 exon alone and pathology type in biopsy sample.

近年来，随着全球工农业技术的发展、大气污染加重、人口老龄化加剧，肺癌的发病率和死亡率均呈上升趋势^[1, 2]^。肺腺癌发病率已超过鳞状细胞癌，由于肺腺癌的多样性和异质性，对放化疗带来一定的影响，患者的生存率有待进一步提高。基因突变技术检测及靶向药物治疗的开展，为肺腺癌的治疗带来了光明，使肿瘤的个体化治疗成为可能。

表皮生长因子受体（epidermal growth factor receptor, EGFR）是一种具有酪氨酸激酶活性的糖蛋白，通过自身磷酸化，调节细胞生长。如果EGFR过表达和突变及信号转导通路异常，将会导致细胞生长失控，形成肿瘤^[3]^。靶向药物吉非替尼、厄洛替尼、阿法替尼等作为EGFR酪氨酸激酶抑制剂有可能通过抑制下游信号异常传导，对*EGFR*敏感性突变形成抑制，为肺癌患者带来希望^[4-6]^。因此*EGFR*基因突变检测备受关注。

手术标本因能提供更全面的组织学形态而更为全面精准地体现腺癌的分化特点，但多数肺癌患者为晚期，活检标本是其唯一可能获得的组织学证据，肺腺癌的异质性在活检标本中是否会导致组织学诊断及*EGFR*基因检测的较大差异，对指导临床是否带来了困扰，因此本研究对不同标本类型的肺腺癌分别统计，观察*EGFR*基因突变与肿瘤的生长方式、分化程度关系。进一步分析*EGFR*基因突变与肺腺癌病理分型及标本类型的关系，为临床检测方案的不断优化和药物治疗提供理论依据。

## 1 材料与方法

### 1.1 材料

收集北京大学第三医院病理科2011年1月-2016年10月肺活检标本173例[粘膜活检、肺穿刺、支气管内超声引导针吸活检术（endobronchial ultrasound-guided transbronchial needle aspiration, EBUS-TBNA）标本]及肺手术切除标本163例（楔形肺切除、肺叶切除标本）石蜡包埋组织，并经病理诊断证实为肺腺癌的病例，行*EGFR*基因检测[基因测序法及突变扩增阻滞系统（amplification refractory mutation system, ARMS）]，按2015年世界卫生组织（World Health Organization, WHO）肺腺癌分型标准对选取的病例进行主要分型确认（贴壁型、腺泡型、乳头型、微乳头型、实体型），鉴于肺腺癌的高度异质性，以占比例最多者为主要分型进行归组。手术标本年龄为29岁-83岁，中位年龄为63岁，男性56例，女性107例；活检标本年龄28岁-84岁，中位年龄为65岁，男性75例，女性98例。分别对手术切除标本和活检标本进行统计，比较两种标本中EGFR基因突变与病理分型的关系是否一致。

### 1.2 方法

手术标本EGFR检测采用荧光PCR Sanger测序法，使用QIAamp DNA FFPE Tissue Kit（#56404）从4片-6片石蜡组织中提取肿瘤的基因组DNA，并以其为模板扩增*EGFR*基因第18-21外显子。扩增条件为预变性95 ℃ 3 min，循环条件为95 ℃变性30 s，56 ℃退火30 s，72℃延伸30 s，共40个循环，72 ℃终末延伸5 min。扩增产物经2%琼脂糖凝胶电泳确定片段大小和产物丰度后，纯化并经ABI 3500基因分析仪进行Sanger测序。测序结果经Alignment软件与Genbank中的EGFR参比序列比对，并利用软件ABI quence Scanner v1.0分析测序峰图，突变病例均经反向测序证实。活检标本采用ARMS荧光PCR法（步骤参见艾德生物试剂盒），分析肺腺癌手术标本和活检标本中肺腺癌类型和*EGFR*突变的情况。

### 1.3 统计学方法

使用SPSS 13.0统计软件对数据分析，各组统计采用行乘列表卡方检验，组间差异采用四格表χ^2^检验，统计数据采用*Pearson*卡方值、*Fisher*确切概率，*P* < 0.05为差异有统计学意义。

## 2 结果

### 2.1 肺腺癌手术切除标本和活检标本中*EGFR*基因突变率及与年龄性别的关系

163例的肺腺癌手术切除标本和173例活检标本中*EGFR*总突变率没有差异（*P* > 0.05）。两组标本中女性的*EGFR*突变率均明显高于男性（*P* < 0.05）。手术标本中60岁以上患者的*EGFR*突变率明显低于60岁以下（*P* < 0.05），而活检标本中*EGFR*突变与年龄无关（*P* > 0.05）（表 1，表 2）。

**1:** 肺腺癌手术和活检标本中*EGFR*基因突变率及与年龄性别的关系 Relationship between the *EGFR* mutation rate and age, gender in lung adenocarcinoma of surgical removal and biopsy specimens

	*n*	EGFR		*χ*^2^	*P*
		Mutation[*n*(%)]	Wild[*n*(%)]		
Surgical removal specimen	163	102(62.58)	61(37.42)		
Gender				4.242	0.039
Male	56	29(51.79)	27(48.21)		
Female	107	73(68.22)	34(31.78)		
Age(yr)				4.105	0.043
< 60	70	50(71.43)	20(28.57)		
≥60	93	52(55.91)	41(44.09)		
Biopsy specimen	173	114(65.90)	59(34.10)		
Gender				9.298	0.002
Male	75	40(53.33)	35(46.67)		
Female	98	74(75.51)	24(24.49)		
Age(yr)					> 0.05
< 60	68	42(61.76)	26(38.24)		
≥60	105	72(68.57)	33(31.43)		
EGFR: epidermal growth factor receptor.					

**2:** 活检标本不同性别中*EGFR*突变与年龄关系 Relationship with the *EGFR* mutation rate and age in different gender of biopsy sample

Gender	Age(yr)	*n*	EGFR		*P*
			Mutation[*n*(%)]	Wild[*n*(%)]	
Male	< 60	31	18(58.06)	13(41.94)	> 0.05
	≥60	44	22(50.00)	22(50.00)	
Female	< 60	37	24(64.86)	13(35.14)	> 0.05
	≥60	61	50(81.97)	11(18.03)	

### 2.2 *EGFR*基因突变的肺腺癌手术切除标本中主要病理分型的构成比及其与外显子的关系

手术标本肺腺癌中*EGFR*基因突变的102例中，腺泡型所占比例最高，其次是贴壁型和乳头型，实体型则比例最少，未见微乳头型。

*EGFR*突变包括18、19、20、21外显子突变。19、21外显子单独突变最多，故对病理亚型及19、21外显子单独突变进行统计，有统计学意义，组间两两比较，21外显子突变在贴壁型较其他两型高（*P* < 0.05），19外显子突变在乳头型较贴壁型高（*P* < 0.05）。腺泡型和乳头型比较，19、21突变无统计学意义（表 3，表 4）。

**3:** *EGFR*突变肺腺癌102例手术标本中主要病理分型的构成及其与外显子的关系 Relationship with the major pathology type and the exon of 102 cases of surgical specimen of lung adenocarcinoma with *EGFR* mutation[*n*(%)]

Pathological type^*^	*n*	Exon 18	Exon 19	Exon 20	Exon 21	Exons 20, 21	Exons 19, 21	Exons 18, 19	Exons 18, 20	Exons 19, 20
Total	102	1(0.98)	45(44.12)	3(2.94)	44(43.14)	1(0.98)	3(2.94)	2(3.57)	2(3.57)	1(1.79)
Lepidic	24	0	4(16.67)	2(8.3)	17(70.83)	0	1(4.17)	0	0	0
Acinar	56	1(1.79)	26(46.43)	0	21(37.5)	1(1.79)	2(3.57)	2(3.57)	2(3.57)	1(1.79)
Papillary	18	0	12(66.67)	1(5.56)	5(27.78)	0	0	0	0	0
Micropapillary	0	0	0	0	0	0	0	0	0	0
Solid	4	0	3(75.00)	0	1(25.00)	0	0	0	0	0
^*^: the pathology type was the major in the case.										

**4:** *EGFR*突变的肺腺癌手术标本中主要病理分型与19、21外显子单独突变的关系 Relationship between the major type and the mutation alone of the exon 19 and 21 in the surgical specimen [*n*(%)]

Pathology type^*^	*n*	Exon 19	Exon 21	*χ*^2^	*P*
Lepidic^a^	21	4(19.05)	17(80.95)		
Acinar^b^	47	26(55.32)	21(44.68)	11.452	0.003
Papillary^c^	17	12(70.59)	5(29.41)		
The number of micropapillary and solid was too small and unable to statistics. ^a^ *vs* ^b^: *χ*^2^=7.746, *P*=0.005; ^a^ *vs* ^c^: *χ*^2^=10.238, *P*=0.001; ^b^ *vs* ^c^: *P* > 0.05. ^*^: the pathology type was the major in the case.					

### 2.3 *EGFR*基因突变的肺腺癌活检标本中主要病理分型的构成比及其与外显子的关系

活检标本肺腺癌中*EGFR*突变的114例中，腺泡型所占比例最高，其次是贴壁型、乳头型、微乳头与实体型最少。

*EGFR*突变包括18、19、20、21外显子突变，其中19、21外显子单独突变最多。不同病理分型中，19、21外显子突变均无明显差异（*P* > 0.05）（表 5，表 6）。

**5:** *EGFR*突变肺腺癌114例活检标本中主要病理分型的构成及其与外显子的关系 Relationship with the major pathology type and the exon of 114 cases of biopsy specimen of lung adenocarcinoma with *EGFR* mutation [*n*(%)]

Pathology type^*^	*n*	Exon 18	Exon 19	Exon 20	Exon 21	Exons 20, 21	Exons 19, 21	Exons 18, 19	Exons 18, 20	Exons 19, 20
Lepidic	30	0	14(46.67)	2(6.67)	11(36.67)	0	2(6.67)	0	1(3.33)	0
Acinar	55	0	24(43.64)	0	29(52.73)	2(3.64)	0	0	2(3.57)	1(1.79)
Papillary	13	0	7(53.85)	0	5(38.46)	1(7.69)	0	0	0	0
Micropapillary	5	0	2(40.00)	0	3(60.00)	0	0	0	0	0
Solid	11	0	6(54.55)	0	5(45.45)	0	0	0	0	0
*: the pathology type was the major in the case.										

**6:** *EGFR*突变的肺腺癌活检标本中主要病理分型与19、21外显子单独突变的关系 Relationship between the major type and the mutation alone of the exon of 19 and 21 in the biopsy specimen [*n*(%)]

Pathology type^*^	*n*	Exon 19	Exon 21	*P*
Lepidic	25	14(56.00)	11(44.00)	
Acinar	53	24(45.28)	29(54.72)	> 0.05
Papillary	12	7(58.33)	5(41.67)	
Micropapillary and solid	16	8(50.00)	8(50.00)	
*: the pathology type was the major in the case.				

### 2.4 *EGFR*基因突变的肺腺癌手术和活检标本病理分型比较

*EGFR*突变的肺腺癌102例手术标本中，腺泡型占54.9%，贴壁型占23.53%，乳头型占17.65%，实体型占3.9%。而114例的活检标本中腺泡型占48.25%，贴壁型占26.32%，乳头型占11.4%，微乳头和实体合并组中占14.4%。经统计*EGFR*基因突变的肺腺癌手术和活检标本中主要病理组织分型构成比明显不同（χ^2^=8.040, *P*=0.045）（表 7）。

**7:** 肺腺癌手术和活检标本中主要病理分型构成比的比较 Comparison with the constitution ration of major pathology type of lung adenocarcinoma in surgical and biopsy specimen [*n*(%)]

	*n*	Leidic	Acinar	Papillary	Micropapillary	Solid	*χ*^2^	*P*
Surgical	102	24(23.53)	56(54.90)	18(17.65)	0	4(3.90)	8.040	0.045
Biopsy	114	30(26.32)	55(48.25)	13(11.4)	5(4.39)	11(9.65)		
Micropapillary and solid being included in the poor differentiation and too little, were combined in statistic(the pathology type was the major in the case).								

## 3 讨论

肺腺癌的显著特点是它的异质性和混合性，2015版WHO相对于2004版的一个区别在于取消了混合型腺癌这一概念，代之以更加精细的分型，并以5%的比例递减进行报告，更加体现了肺腺癌的异质性，人们对肺腺癌的基因突变认识也只是冰山一角。目前*EGFR*基因突变是在肺腺癌最常见，由细胞外域、细胞内域、跨膜区三部分组成^[7]^，细胞内域含有酪氨酸激酶^[8]^，通过自身磷酸化，进一步激活与细胞增殖、凋亡有关的通路，调节细胞生长、分化和增殖。如果EGFR过表达和突变及信号转导通路异常，将导致细胞DNA复制及细胞分裂过度，导致细胞生长失控和恶性化，形成肿瘤。EGFR在非小细胞肺癌发病中，具有促进肿瘤发生、发展及转移的作用^[9]^。

本研究中163例的肺腺癌手术标本中，*EGFR*基因突变率为62.58%。173例的肺腺癌活检标本中，*EGFR*基因突变率为65.9%，两组标本*EGFR*突变率没有显著差异（*P* > 0.05）。肺腺癌手术标本中女性*EGFR*突变率（42.77%）明显高于男性（23.12%）。肺腺癌活检标本中女性*EGFR*突变率（44.79%）明显高于男性（17.79%）（*P* < 0.05），两组标本中EGFR女性突变率均明显高于男性。

Shi等^[10]^报道在亚洲东南地区肺腺癌中，女性为*EGFR*突变率为61.1%明显高于男性（44%），不同地区*EGFR*突变也有差异，突变率最高为越南64.2%（77/120），最低为印度22.2%（16/72）。其他国家在47.2%-62.1%之间（中国大陆为50.2%，香港为47.2%，印度为22.2%，菲律宾为52.3%，台湾为62.1%，泰国为53.8%，越南为64.2%）。本研究中*EGFR*突变稍高，和台湾、越南相差不多。Han等^[11]^报道国内276例的肺的非小细胞腺癌中，*EGFR*基因突变率为55.8%，女性的突变率明显高于男性，和年龄没有明显相关关系。Liang等^[12]^报道国内肺腺癌中*EGFR*突变率为63.9%，女性明显高于男性，突变率与年龄无关。诸多报道中女性突变高于男性，本研究与此一致。而Zhang等^[13]^发现东亚地区女性非吸烟者手术切除的肺腺癌标本中*EGFR*的突变率为76.2%，年龄越大突变率越高。而本研究手术标本中，年龄越大，*EGFR*突变率越低，与其他报道结果不同。而活检标本中*EGFR*突变与年龄无关，男性女性标本中*EGFR*突变均与年龄无关。由此可见对于EGFR与年龄的关系尚不明了。有待进一步探讨。

手术标本中*EGFR*突变包括18、19、20、21外显子突变。19、21外显子单独突变最多（分别为44.12%和43.14%），21外显子突变在贴壁型较其他两型高（*P* < 0.05），19外显子突变在乳头型较贴壁型高（*P* < 0.05）。实体型中虽然19外显子比21外显子突变率高，但病例数较少，无法统计。提示在不同外显子突变中病理分型不同。19外显子可能在低中分化腺癌中突变较高，而21外显子在高分化腺癌中突变较高（贴壁型为高分化，腺泡型和乳头型为中分化，微乳头和实体型为低分化）。目前，19、21外显子突变被认为是两种独立的疾病，对EGFR酪氨酸激酶抑制剂反应不同，Zhu等^[14]^认为19外显子突变的患者对吉非替尼反应比21外显子突变者明显。Sordella等^[15]^报道，19和21外显子突变能导致EGFR分子自身磷酸化的位点不一样，而导致其下游的信号通路不同，比如，相对于缺失突变，L858R突变中的845密码子编码的酪氨酸残基表现出高度磷酸化的状态。这可能是19外显子突变肺癌患者相对21外显子突变肺癌患者对酪氨酸激酶抑制剂（tyrosine kinase inhibitors, TKIs）药物反应率较高，应用TKIs药物后预后较好的原因。由此建立了组织学分型与EGFR状态及TKI治疗三者的关系。

*EGFR*基因突变的肺腺癌手术标本102例中，主要病理分型腺泡型所占比例最高（54.9%），其次是贴壁型（23.53%）和乳头型(17.65%)，实体型则比例最少（3.9%）。*EGFR*突变的肺腺癌活检标本114例中，腺泡型所占比例最高（48.25%），其次是贴壁型（26.32%）、乳头型（11.4%），微乳头与实体型最少（14.04%）。手术和活检标本比较，主要病理分型构成比不同。活检标本肺腺癌中*EGFR*基因突变的114例中，19、21外显子突变与主要病理分型无相关关系。朱佩^[16]^发现在气管镜活检标本中*EGFR*基因突变与肺腺癌的分化无明显相关。本研究与此结果一致。由于肺腺癌病理分型多样性，活检标本可能只反映了肿瘤的部分组织学类型，比较局限，不能反应全貌，不能替代主要组织学分型，故不能得出手术标本表现出的组织学分型与EGFR之间的关联性。因此对于评判病理分型与基因突变的关系时，活检标本可能没有手术标本更能表现出的相关性。此外，活检标本EGFR检测使用的是ARMS方法，手术标本使用的是PCR Sanger测序法，由于方法差异引起的误差不可避免，因此尚需统一方法进一步研究。

本研究中看到了19与21外显子有不同的组织学形态特点，但是其中是否有更深的机制，尚待进一步研究。肺癌靶向治疗虽然如火如荼开展，但还需要相关理论作为支撑，对于*EGFR*基因相关通路中不同外显子的突变疗效不同的具体机制，还需进一步探讨，尤其是肺腺癌异质性较大，同一患者可能多种病理分型，以及手术和活检标本*EGFR*基因检测的差异，诸多问题需要关注，因此对于肺癌的靶向治疗及预后评估需要很长的路要走。
